# Supplementary material for: Acceptance and compliance with micronutrient powder (MNP) among children aged 6–23 months in northern Nigeria
Source: PLOS Glob Public Health. 2022 Oct 17;2(10):e0000961. doi: 10.1371/journal.pgph.0000961 (PMC10022258; doi:10.1371/journal.pgph.0000961)
Supplement: S1 Dataset — (ZIP) [file pgph.0000961.s008.zip › S8_Dataset/Spot Checks.zip!/Spot Checks/080715_K_J_BK_2_SC.b.pdf]

SPOT CHECK RECORDING FORM

State: Kebbi LGA: Jega Community: Arausaya

Sex of index child: F Age of index child (in months): 21

Spot Check Information

1. **Date of MNP distribution to household** (circle option below based on LGA name above)**:**
   - **Kebbi:** BirninKebbi&Jega LGA: **June 16**
   - **Kebbi:** Danko-wasagu: **June 17**
   - **Adamawa:**Song, Ganye: **June 18**
   - **Adamawa:**Guyuk **June 19**
2. **Date of Spot Check Observation: 8/7/15**
3. **Number of sachets remaining**(Ask the caregiver to show you and count the specific number of sachets remaining)**:**

38

4. **Number of sachets that should remainusing one MNP sachet per day** (Analyst only)**:**

39

5. **Number of sachets remaining / number of sachets should be remaining**(Analyst only)**:**

   ______38___ / ____39_____ = ____97.4_____ %

Probes

Question 1. You have **XX** MNP sachets remaining. Explain how easy or difficult it was to use the MNP product on a daily basis for your child 6 – 23 months? (Circle one answer)

Very Difficult **Difficult** **Easy** **Very Easy**

Question 2. Describe any specific challenges that you faced that made the MNP difficult to use on a daily basis?

The only challenge is that the girl noticed that I usually remove a small portion of kunu and mix with MNP, and she started refusing it, so I have to change the food

Question 3. Describe specific aspects of the MNP product that you or your child enjoyed that made it easier to use on a daily basis?

The child has become healthier and eats a lot now, she was suffering from eye problem but it has stopped since I started giving her the product. She is strong now

Question 4. What questions do you have about the MNP based on your experience using it?

What other food can I add it too, since she is refusing the kunu?

Question 5. What specific recommendations do you have that we can do as a program to make the MNP easier to use on a daily basis?

I like it and wish other children will be give too, free of charge and enough quantity.
